# IncF Plasmids Are Commonly Carried by Antibiotic Resistant* Escherichia coli* Isolated from Drinking Water Sources in Northern Tanzania

**DOI:** 10.1155/2016/3103672

**Published:** 2016-03-24

**Authors:** Beatus Lyimo, Joram Buza, Murugan Subbiah, Sylivester Temba, Honest Kipasika, Woutrina Smith, Douglas R. Call

**Affiliations:** ^1^Nelson Mandela African Institution of Science and Technology, 447 Arusha, Tanzania; ^2^Paul G. Allen School for Global Animal Health, Washington State University, Pullman, WA 99164, USA; ^3^One Health Institute, School of Veterinary Medicine, University of California, Davis, CA 95616, USA

## Abstract

The aim of this study was to identify the replicon types of plasmids, conjugation efficiencies, and the complement of antibiotic resistance genes for a panel of multidrug resistant* E. coli* isolates from surface waters in northern Tanzania. Standard membrane filtration was used to isolate and* uidA* PCR was used to confirm the identity of strains as* E. coli*. Antibiotic susceptibility was determined by breakpoint assay and plasmid conjugation was determined by filter-mating experiments. PCR and sequencing were used to identify resistance genes and PCR-based replicon typing was used to determine plasmid types. Filter mating experiments indicated conjugation efficiencies ranged from 10^−1^ to 10^−7^. Over 80% of the donor cells successfully passed their resistance traits and eleven different replicon types were detected (IncI1, FIC, P, FIIA, A/C, FIB, FIA, H12, K/B B/O, and N). IncF plasmids were most commonly detected (49% of isolates), followed by types IncI1 and IncA/C. Detection of these public health-relevant conjugative plasmids and antibiotic resistant traits in Tanzanian water suggests the possible pollution of these water sources from human, livestock, and wild animal wastes and also shows the potential of these water sources in the maintenance and transmission of these resistance traits between environments, animals, and people.

## 1. Introduction

Increased mortality and morbidity due to antibiotic treatment failure make antimicrobial resistance (AMR) one of the 21st century's major global public health challenges [1]. Overuse and misuse of antibiotics are considered major reasons for the emergence of resistant bacteria in many low-income countries [2, 3]. Antibiotic resistance has been documented for enteric bacteria from various water sources and these water sources could facilitate dissemination of resistant bacteria to a wider community of people and animals [4]. This is particularly true for low-income countries like Tanzania where water sources are frequently shared between animals and people [5, 6]. For example, a report from Kenya reported a high prevalence of antibiotic resistance* E. coli* from water and fish in Lake Victoria [ampicillin (64%), tetracycline (76%), and cotrimoxazole (80%)] [7] where untreated water is consumed routinely.

Tanzanian hospitals have reported a high proportion (80%–90%) of clinical* E. coli* isolates that are resistant to antibiotics such as ampicillin, cotrimoxazole, tetracycline, gentamicin, and amoxicillin/clavulanic acid. These bacteria infect people within a healthcare system where, in most cases, there are no laboratory diagnostics to guide antibiotic treatment [8–10]. Another study reported a high number of antibiotic resistant* E. coli*, possessing resistance to cephalosporins, from free-range buffalo, zebra, and wildebeest [11]. These animals were located in mixed grazing areas with potential contact with people and livestock. Contaminated water was suspected as the source of resistant bacteria found in these wild animals [11]. Contaminated water most likely plays a role in the dissemination of antibiotic resistant bacteria and the probability of transmission likely increases when people and animals use that water.

Antimicrobial resistance (AMR) genes are transferred to other bacteria, sometimes at the species level, by horizontal gene transfer (transduction, transformation, and conjugation). Plasmid-mediated horizontal transfer of multidrug resistance between different bacteria is a major concern because this contributes to the evolution and emergence of antibiotic resistant bacteria in the environment [12, 13].

For the last decade, polymerase chain reaction-based replicon typing (PBRT) has been used to identify major plasmid types found in Enterobacteriaceae, including incompatibility (Inc) groups (HI2, HI1, I1-*γ*, X, L/M, N, FIA, FIB, FIC, W, Y, P, A/C, T, K, and B/O) [14, 15]. Plasmids belonging to group IncF frequently harbor *bla*_CTX-M-15_ that is often associated with *bla*_TEM-1_, bla_OXA-1_, and* aac(6*′*)-Ib-cr* resistance genes [16]. Replicon groups IncA/C and l1 are frequently associated with Enterobacteriaceae and harbor multiple resistance genes including resistance for extended-spectrum cephalosporins and carbapenems [17–19].

In northern Tanzania, surface water such as rivers and ponds is often shared between animals and people on daily basis. Consequently, these water sources become polluted with human and animal excreta and might harbor antibiotic resistant enteric bacteria. Consumption of water containing these bacteria is likely to increase the risk that antibiotic resistant and pathogenic bacteria will be transmitted. Nevertheless, to date, no studies have been conducted in Tanzania to determine if drinking water represents a risk factor for transmission of antibiotic resistant bacteria to people and animals. The objective of this study was to characterize the replicon types of plasmids that harbor drug resistant traits, their conjugation efficiencies, and the complement of antibiotic resistance genes for a panel of multidrug resistant* E. coli* isolates that were obtained from drinking water sources in northern Tanzania.

## 2. Methods

### 2.1. Study Design

Convenience sampling was used to collect water samples between March and August 2014. Each source was visited twice and one sample was collected from each source per visit (in Tanzania, March is the rainy season and August is during dry season). Sample locations included the Kilimanjaro Region (Moshi Municipal, Moshi Rural, and Hai Districts), the Arusha Region (Arusha City, Arumeru, Longido, and Monduli Districts), and the Manyara Region (Simanjiro and Babati Districts). A convenience approach was used to select sampling sites with appropriate permission from local authorities. Water samples from ponds were collected from localities near Maasai villages. All sites, including streams, may have been impacted by people and wildlife. We also collected opportunistic samples from taps and wells.

### 2.2. Isolation and Identification of* E. coli*

Water samples were collected in 500 mL sterile bottles and were transported in cooler boxes with ice packs to the laboratory for processing within 6 h of collection. Out of 500 mL, 100 mL water samples were analyzed using a standard membrane filtration technique with minor modifications [20]. Following filtration, each filter membrane was placed on a chromogenic selective agar plate (HiCrome* E. coli* Agar, HiMedia Laboratories Prt. Ltd., Mumbai, India). The agar plates were initially incubated at 37°C for 4 h, followed by incubation for 16–22 h at 44°C. Plates produced 1–200 CFU of which individual colonies were subcultured to ensure purity for further characterization. The identity of* E. coli* isolates was confirmed using a PCR genotyping test that detects the presence of the* uidA* gene [21].

Antibiotic break point assays were used to determine the resistance profile of each* E. coli* isolate against a panel of important antibiotics. MacConkey (MAC) (Thermo Oxoid Remel) agar plates with each antibiotic at their CLSI recommended minimum inhibitory concentrations [22] (Table 1) were used to perform the break point assays [23].* E. coli* strains K-12 (negative control; susceptible to all antibiotics tested) and H4H* E. coli* (positive control; resistant to all antibiotics tested) were used as reference strains for antibiotic susceptibility testing.

### 2.3. Plasmid Characterization

A set of 31* E. coli* isolates that were susceptible to nalidixic acid and resistant to more than 2 antibiotics tested were chosen for this study. Filter-mating experiments were performed to determine the conjugation rates of plasmids with the nalidixic acid susceptible MDR (wild-type)* E. coli* isolates as donors and a plasmid-free recipient strain (*E. coli* K-12, nalidixic acid resistant, Nal^r^) as recipient as described earlier [23, 24] with minor modifications. Briefly, single colonies of* E. coli* K-12 and potential donor strains were grown separately overnight in LB medium (Luria-Bertani medium; Difco*™* LB Broth Lennox, Sparks, MD, USA) at 37°C. Equal quantities (10 *μ*L) of overnight cultures of donor and recipient strains were added on top of a nitrocellulose (~1 cm^2^) membrane overlaid on LB agar with no antibiotics. After 24 h of incubation at 37°C, cells from the membrane were suspended in 500 *μ*L of sterile phosphate-buffered saline (PBS, pH 7.0) and spread onto LB agar plates containing 32 *μ*g/mL nalidixic acid (Sigma-Aldrich) and another antibiotic to which the donor cells were resistant (Table 2). Colonies that grew on these selective agar plates were considered transconjugants. The conjugation efficiency of plasmid was calculated by dividing the number of transconjugants by the number of donor cells. Transconjugants were screened for their donor's antibiotic resistance phenotypes and presence of* tet*(A),* tet*(B), *bla*_TEM-1_, *bla*_SHV_, and *bla*_CTX-M_ genes [25, 26]. CTX-M grouping (group 1, group 2, and group 9) was further evaluated for all CTX-M positive isolates. All CTX-M* E. coli* isolates were positive for CTX-M group 1 and the PCR products were subsequently sequenced by Functional Bioscience (Madison, WI). Sequencher (ver 5.0) software was used to process sequence traces, and the final sequences were analyzed with CLC Genomic Workbench 7.0.2 (CLC Bio Aarhus, Denmark) and compared with the reported sequences from GenBank (http://blast.ncbi.nlm.nih.gov/Blast.cgi).

PCR-based replicon typing was used with genomic DNA of the transconjugants using the methods described by Johnson et al. [15]. Briefly, pellets from 1 mL of overnight culture were resuspended with 200 *μ*L of nanopure water and placed in a heating block at 100°C for 10 min. The lysed suspension was cooled to room temperature and centrifuged briefly to pellet debris. Supernatant was transferred to a new vial and stored at −80°C until ready for testing against 18 different sets of primers [15] that were grouped into three multiplex primer panels [15]. The following PCR conditions were used: 5 min at 94°C, 30 cycles of 30 s at 94°C, 30 s at 60°C, and 90 s at 72°C, and a final extension of 5 min at 72°C. The amplified PCR products were visualized using 1.5% Tris-acetate-EDTA agarose gel containing 0.2 *μ*g/mL ethidium bromide alongside a 1 kb ladder (Gene ruler 1 Kb, Life Technologies).

## 3. Results

Thirty-one MDR isolates were selected and used as donors to test if the resistance determinants were transferrable to recipient* E. coli* isolates by conjugation. Of these, antibiotic resistance traits were successfully transferred by 25 isolates with conjugation efficiencies ranging from 10^−1^ to 10^−7^ (Table 2). IncF plasmids were attributable to the highest conjugation efficiency 1.8 × 10^−1^. Importantly, over 80% of the donor cells successfully passed a “penta-resistant” phenotype that included resistance to ampicillin, streptomycin, sulfamethoxazole, tetracycline, and trimethoprim. PCR testing of transconjugants showed that* tet*(A) was most commonly associated with conjugative plasmids (33%) followed by *bla*_TEM-1_ (24%),* tet*(B) (17%), *bla*_CTX-M_ (8%), and bla_SHV-1_ (0%).

A total of 11 replicon types were detected among the 31 MDR isolates (Table 3). IncF replicon types (IncF IA, IB, IC, and IIA) were predominant (49%) and were mainly associated with* E. coli* isolates that were resistant to ampicillin, streptomycin, sulfonamide, tetracycline, and trimethoprim. Replicon types IncX, IncW, IncL/M, IncY, IncHI1, IncT, and Inc K were not detected. Replicon types N, H12, FIB, and FIA were associated with *bla*_CTX-M-15_ and resistance to ampicillin, ceftazidime, streptomycin, sulfamethoxazole, tetracycline, and trimethoprim. After conjugation, one recipient was positive for four different replicons (I1, FIB, FIA, and K/B).

## 4. Discussion

Plasmid-mediated horizontal transfer of multidrug resistance traits plays a key role in the dissemination of antimicrobial resistance around the world [27]. Our study shows that* E. coli* isolated from Tanzanian water sources harbor multiple plasmids belonging to major plasmid replicon types such as IncF, A/C, l1, and N. Most of these plasmids were associated with transfer of antibiotic resistance traits via conjugation with rates that varied between 10^−1^ and 10^−7^ using filter-mating assays. Moreover, these plasmids harbor multiple antibiotic resistance genes that are associated with plasmid replicon types such as IncF, A/C, N, l1, H12, and B/O. Studies show that plasmid-mediated horizontal gene transfer occurs within and between* E. coli* and* Pseudomonas* isolated from sewage and lake water [13]. Given the presence of resistant* E. coli* in biologically contaminated water from Tanzania, it is likely that their presence contributes to the long-term persistence of resistance traits in people and animals who share these water resources.

Among the 11 replicon types found, IncF group plasmids were detected more frequently than other tested groups. This is in accordance with previous studies where IncF plasmids were found predominantly in* E. coli* from clinical samples (rectal samples, gastric aspirate samples, and vaginal sample) [28, 29] and in* E. coli* from people (feces and UTI patients) and poultry (fecal swab) [15]. IncFIB was the most frequently detected (16%) replicon type, similar to what has been reported for* E. coli* isolates collected from fecal samples of healthy people and cattle in Nigeria [30]. The overall proportion of IncF-positive* E. coli* was 49%, which is lower than that observed in Germany (71%) [31] but higher than that observed in* E. coli* isolates from fecal samples of healthy people and food animals in Switzerland (45%) [27]. IncF type plasmids have a “narrow” host range although they are well adapted to* E. coli* and are frequently associated with the presence of* tet*(A), *bla*_TEM-1_, and *bla*_CTX-M_ [31, 32]. In this study, plasmid type IncF was associated with* tet*(A), *bla*_TEM-1_, and *bla*_CTX-M-15_.

The CTX-M-15 *β*-lactamases are disseminated worldwide and are usually located in the conjugative plasmids [33]. Detection of this trait in* E. coli* isolated from water sources is a public health concern because CTX-M-15 *β*-lactamases are commonly associated with urinary tract infections [30]. Another study from a hospital in Tanzania found CTX-M-15 in* Klebsiella pneumoniae* that can be associated with neonatal sepsis [34]. In this study, detection of CTX-M-15 in* E. coli* from water suggests a possible contamination of human, livestock, and wild animal excreta and thus consumption of this untreated water is clearly a potential risk for transmission back to people [35].

The IncI1 plasmid type was the second most prevalent replicon type (13%) and these plasmids also harbor multiple resistance genes.* E. coli* can reportedly maintain IncI1 plasmids without antibiotic selection pressure and with little or no apparent fitness cost to the host bacterium [36]. Importantly, it is also a conjugative plasmid commonly detected in* E. coli* recovered from humans and animals with the conjugation efficiencies ranging between 10^−2^ and 10^−7^ [15, 16, 28, 34]. The IncI1 plasmid carrying *bla*_CTX-M-15_ and *bla*_TEM-1_ has been associated with the recent 2011 outbreak of* E. coli* O104 in Germany [37]. In addition, bacteria carrying lncl1 plasmids were responsible for community and hospital acquired infections [38, 39].

IncA/C plasmids are typically larger (~150 kb) than others with lower conjugation efficiencies [24, 40, 41]. About 10% of* E. coli* isolates from our water samples harbored IncA/C plasmids and these were associated with resistance to ampicillin, streptomycin, sulfonamide, tetracycline, and trimethoprim. Importantly, IncA/C plasmids can harbor a large number of antimicrobial resistance genes and the broad-host spectrum coupled with an ability to spread via conjugation transfer within bacteria communities means that they can transfer an arsenal of resistance traits to pathogens of people and animals [40, 42, 43]. Isolation of* E. coli* with IncA/C from environmental water samples that are consumed by humans and animals on daily basis is a major public health concern. The replicon typing methods have some pitfalls including the obvious inability to detect unknown replicons [15]. For example, 14 isolates in the current study transferred their resistance phenotypes to the recipients' cells but no plasmid replicon was detected. Detection of these public health important conjugative plasmids and antibiotic resistant traits in Tanzanian water suggests the possible pollution of these water sources from human, livestock, and wild animal wastes and also shows the potential of these water sources in the maintenance and transmission of these resistance traits between environment, animals, and people. Therefore, appropriate intervention strategies should be identified and implemented to reduce the water pollution.

## Figures and Tables

**Table 1:** Antibiotic concentration tested against *E. coli* from surface waters in northern Tanzania.

Name	Code	Source	Concentration (*µ*g/mL)
Amoxicillin/clavulanate potassium	Amx/Clv	MP Biomedicals, Illkirch, France	32/16
Ampicillin	Amp	Fisher Scientific, Fair Lawn, New Jersey	32
Ceftazidime	Ceftaz	Sigma-Aldrich	16
Chloramphenicol	Chlo	Sigma-Aldrich	32
Ciprofloxacin	Cip	Sigma-Aldrich	4
Kanamycin	Kan	Sigma-Aldrich	64
Streptomycin	Str	Sigma-Aldrich	16
Sulfamethoxazole	Sul	MP Biomedicals	512
Tetracycline	Tet	GTS, San Diego, CA	16
Trimethoprim	Tri	MP Biomedicals	16

**Table 2:** Sample collection sites, phenotypes of donor and transconjugants, conjugation efficiency, resistance genes, and associated plasmid replicons.

District	Resistance phenotypes of donors	Abx used for selection	Resistance genes in transconjugants	Conjugation efficiency	Replicon type in transconjugants	Resistance phenotypes of transconjugants
Arusha urban	StrSulTri	Str	ND	—	I1	Str
	StrTri	Str	ND	—	FIC	Str

Moshi urban	AmpStrSulTetTri	Tet	*tet*(B), TEM-1	5.8 × 10^−3^	ND	AmpStrSulTetTri
	AmpStrSulTetTri	Amp	*tet*(A)	3.83 × 10^−3^	P, FIIA	AmpStrSulTetTri
	AmpStrSulTet	Amp	*tet*(A)	9.88 × 10^−1^	ND	AmpStrSulTet
	SulTetTri	Tet	*tet*(A)	9.93 × 10^−1^	ND	SulTetTri
	StrTetTri	Tet	*tet*(B)	2.00 × 10^−7^	ND	StrTetTri
	AmpStr	Amp	ND	2.7 × 10^−4^	FIIA	Amp

Moshi rural	AmpStrSulTetTri	Amp	tet(A), TEM-1	8.33 × 10^−6^	A/C, P, FIB	AmpStrSulTetTri
	AmpSulTetTri	Tet	*tet*(A)	7.86 × 10^−2^	FIA, FIB	AmpSulTetTri
	AmpStrSulTetTri	Tet	*tet*(A)	2.05 × 10^−7^	A/C	AmpStrSulTetTri

Hai rural	AmpStrSulTetTri	Amp	ND	7.49 × 10^−3^	ND	Amp
	AmpStrSulTet	Amp	ND	—	ND	Amp

Simanjiro rural	AmpCeftazKanStrSulTetTri	Amp	*bla*_CTX-M-15_	6.5 × 10^−2^	N, FIB, FIA	AmpStrSulTetTri
	AmpCeftazChloStrSulTetTri	Amp	*bla*_*C*TX-M-15_	—	N, H12	AmpCeftazStrSul
	AmpStrTetTri	Tet	*tet*(B)	1.08 × 10^−4^	ND	AmpStrSulTet
	AmpSulTetTri	Amp	ND	6.25 × 10^−4^	ND	AmpSul
	TetTri	Tet	ND	—	ND	Tet

Monduli rural	AmpCeftazChloKanStrSulTetTri	Tet	*tet*(A), *bla*_TEM-1_	9.26 × 10^−2^	H12, K/B	AmpCeftazChloKanStrSulTetTri
	AmpStrSulTetTri	Amp	*tet*(A)	3.19 × 10^−4^	ND	AmpSulTet
	AmpStrSulTet	Amp	ND	1.07 × 10^−2^	I1, FIB	AmpSul
	AmpSulTet	Amp	ND	7.91 × 10^−3^	B/O	AmpSulTet
	AmpSul	Amp	ND	2.45 × 10^−2^	ND	AmpSul

Longido rural	AmpStrSulTetTri	Amp	ND	5.31 × 10^−3^	B/O, FIC	AmpSul
	AmpStrSulTri	Amp	ND	8.93 × 10^−4^	FIC	AmpSul
	AmpSulTetTri	Tet	ND	8.99 × 10^−5^	FIC, A/C, FIIA	AmpSulTet
	AmpStrTet	Amp	ND	1.33 × 10^−4^	I1, K/B	AmpStrTet

Arumeru periurban	AmpChloKanSulTetTri	Amp	*tet*(B), *bla*_TEM-1_	8.95 × 10^−2^	I1, FIB, FIA, K/B	AmpKanStrSulTetTri
	AmpChloSulTetTri	Amp	ND	1.66 × 10^−2^	ND	AmpSul
	AmpStrSulTetTri	Amp	ND	5 × 10^−3^	ND	Amp
	AmpSulTetTri	Amp	*bla*_TEM-1_	8.33 × 10^−1^	ND	AmpSulTetTri

Abx, antibiotic; Amp, ampicillin; Ceftaz, ceftazidime; Cip, ciprofloxacin; Chlo, chloramphenicol; Kan, kanamycin; Str, streptomycin; Sul, sulfamethoxazole; Tet, tetracycline; Trm, trimethoprim; ND, not detected.

**Table 3:** Frequency of plasmid replicon types detected from *E. coli* isolates (*n* = 31) from surface waters in northern Tanzania.

Replicon type	Number of isolates	Percent of isolates
FIB	5	16%
FIC	4	13%
I1	4	13%
FIA	3	10%
FIIA	3	10%
A/C	3	10%
K/B	3	10%
P	2	6%
HI2	2	6%
B/O	2	6%
N	2	6%

The total percentage sums to 106 because some isolates were positive for more than one replicon type.
